# Effects of Straw Return and Moisture Condition on Temporal Changes of DOM Composition and Cd Speciation in Polluted Farmland Soil

**DOI:** 10.3390/ijerph191912128

**Published:** 2022-09-25

**Authors:** Guang Yang, Xiangyu Tang, Zhuo Guan, Junfang Cui

**Affiliations:** 1 Key Laboratory of Mountain Surface Processes and Ecological Regulation, Institute of Mountain Hazards and Environment, Chinese Academy of Sciences, Chengdu 610041, China; 2 University of Chinese Academy of Sciences, Beijing 100049, China; 3 State Key Laboratory of Subtropical Silviculture, Zhejiang A&F University, Hangzhou 311300, China

**Keywords:** soil moisture, dissolved organic matter, cadmium, straw, fluorescence parameters

## Abstract

Straw return can improve soil quality and change the mobility and bioavailability of pollutants in soil. Elevated cadmium (Cd) contents in farmland soils were often reported. However, the impacts of straw-derived dissolved organic matter (DOM) on Cd speciation in soil remain poorly understood. In this study, the effects of straw return and moisture condition on temporal changes of DOM composition and Cd speciation in farmland soils were explored through a laboratory incubation experiment. The humified components of DOM were negatively correlated with exchangeable, carbonate-bound, and Fe-Mn oxide-bound Cd (*p* < 0.01), while its protein-like component was negatively correlated with residual Cd (*p* < 0.01). It was found that selected fluorescence parameters could be used to predict temporal changes of Cd geochemical fractions. Straw addition led to increases in soil DOM content during the first three days of the incubation. Flooding should be avoided in the first three days following the straw application to reduce the risk of DOM-facilitated Cd mobilization.

## 1. Introduction

Cadmium (Cd) is a highly toxic heavy metal. Cd pollution in farmland soils has been often reported and attracted much attention in many countries around the world [1,2,3,4]. Straw return is a common agricultural practice for improving soil quality [5]. In 2015, 1.04 billion tons of straw was produced in China, which accounted for about one-third of the global production [6]. The national straw utilization rate in China was approximately 80%, with the incorporation of straw into farmland being its main utilization [7]. The straws returned to the field are mainly rice, wheat, and maize straw [6]. It should be noted that the mobility and bioavailability of Cd in soils may be affected by straw return, but inconsistent findings have been reported [8,9,10,11,12]. For instance, decreases in dissolved Cd in soils were observed upon rice straw amendment [8] while increases in dissolved Cd in soils by wheat straw amendment were found [10]. Such inconsistencies were largely unexplained [12], and the underlying mechanisms of straw return on Cd speciation remain unclear.

Crop straw contains not only various nutrient elements (e.g., nitrogen, phosphorus, and potassium) but also organic compounds (e.g., cellulose, hemicellulose, lignin, protein, and carbohydrates) [13,14]. Mature organic matter of soil matrix and freshly introduced materials (e.g., materials produced from rhizoexduation, biomass/cell lysis, or decomposition of straws or litter) are the two major sources of dissolved organic matter (DOM) in soils [15]. Crop straw-derived DOM comprises various organic compounds, including non-humic biomolecules (e.g., carbohydrates, amino acids, proteins, lignin, organic acids, and fatty acids) and humic substances (e.g., humic and fulvic acids, which are formed after straw decomposition and transformation) [16]. Compared to soil DOM, straw DOM contains a great deal of lower molecular weight compounds (e.g., amino acids, proteins, monosaccharides, polysaccharides, and carbohydrates), which can be leached and degraded after straw application [17,18]. At a later stage of straw decomposition, the DOM released is mainly aromatic compounds [19,20].

DOM contains abundant functional groups (e.g., carboxyl, phenol, and hydroxyl), which can form complexes with Cd [21,22,23]. In a sandy sulfuric soil, the addition of straw DOM was found to rapidly induce redox processes and pH increase [24]. It has been well known that redox potential (Eh) and pH are key environmental factors in controlling the solubility and bioavailability of Cd in soils [25]. Soil Eh can be changed by water management. Flooding leads to decreases in dissolved Cd, as a result of the transformation to CdS or Fe-Mn (oxyhydro) oxide-bound Cd showing low solubility [26,27]. However, the potential role of DOM in meditating the shift of Cd distribution among different geochemical fractions has not been considered or recognized. Few studies have been conducted to reveal the dynamics of DOM and Cd in soils and their interactions during the course of straw decomposition. In this study, it is hypothesized that, following straw application, the quantity and composition of straw DOM vary with time and can be affected by soil moisture conditions, and temporal changes of Cd speciation in soil may be facilitated by DOM.

The objective of this study was to reveal the effects of straw return and moisture conditions on the dynamics of Cd speciation in farmland soils. A laboratory incubation experiment was carried out for Cd polluted soils of paddy-upland farmland, which experienced both saturated and unsaturated conditions and receive the amendment of different types of crop straw. Correlation analysis on time series data across different treatments was conducted to elucidate the relationships of Cd geochemical fractions with DOM components.

## 2. Materials and Methods

### 2.1. Material Collection and Sample Preparation

**Figure 1:**
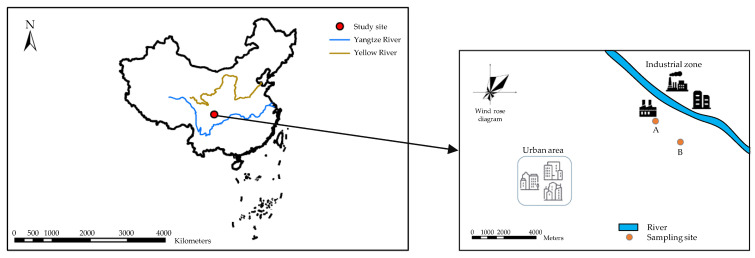
Locations of sampling sites in the downwind area of a phosphorus chemical industrial zone in Shifang, Sichuan, China.

Surface soils (0–20 cm) were collected from paddy-upland rotation farmland sites A and B in Shifang (104°16′ E, 31°10′ N) city, Sichuan Province, China. The soil of both sites belongs to paddy soil, which is the main soil type accounting for 89.7% of farmland in Shifang and was derived from alluvial deposit. Site A was close (20 m) to the boundary wall of a phosphorus chemical industrial zone while site B was 2 km away from the industrial zone (Figure 1). At each site, a 10 × 10 m square sampling plot was established. Five soil samples were taken randomly within the plot and combined into one composite sample [28]. After air drying, gravels and crop residue (diameter > 1 cm) were manually removed from the two composite soil samples. A total of 1000 g of soil sample was ground, passed through a 2 mm nylon sieve, and then stored in a wide-mouth glass bottle before use. Fresh maize straw and rice straw were collected locally from newly harvested unpolluted farmland and found to be Cd-free. The soil pH of the samples from sites A and B was 6.2 and 6.5, respectively. Cd contents of the soil samples from sites A and B were measured to be 6 mg kg^–1^ and 1 mg kg^–1^, respectively. Specifically, the Cd content of the soil sample from site A exceeded the risk intervention value (2.0 mg kg^–1^ for soils with pH greater than 5.5 but not higher than 6.5) for agricultural land in China, according to GB 15618-2018 [29]. These two soil samples were defined as heavily and slightly polluted soil, respectively. After being cut into 1–2 cm sections, the straw samples were washed with ultrapure water and dried at 60 °C for 48 h. Afterwards, the straw samples were ground, passed through a 0.25 mm sieve, and then stored in sealed plastic bags prior to use.

### 2.2. Laboratory Incubation Experiment

A laboratory incubation experiment was conducted at 25 °C to simulate the dynamics of straw decomposition in the soils. A total of 100 g of soil was put into the plastic cup (12 cm in height, 7 cm in diameter; without drainage holes at the bottom). Maize or rice straw was applied at a rate of 2% of the soil weight (oven-dry basis), and a control (without straw addition) was set for each soil. The shredded straw was added in two ways: by placing in a mesh bag buried in soil and by thorough mixing with soil (without mesh bags), in order to reveal the differences between the temporal changes of straw DOM alone in the soil and overall DOM of the soil-straw mixture. Two moisture conditions of incubation were employed in comparison: continuous flooding (with 1 cm overlying water) and 75% of the field capacity. In particular, for the latter treatment, the soil water content was maintained by adding deionized water every 3 days. All the treatments were performed in duplicate. On days 0, 3, 7, 15, 30, 60, and 90, soil and straw samples were taken from the cups, freeze-dried, and then kept in sealed plastic bags before analysis.

Data of the DOC (i.e., water extractable organic carbon using the method described in 2.3) content were fitted using the following first-order non-linear kinetic model (Equation (1)) [30].

(1) $$C_{t}=C_{0}+ae^{-kt}$$

where *C*_t_ is the soil DOC content at time *t* (day) after the commencement of laboratory incubation experiment (g kg^–1^), *C*_0_ is the final stable level of soil DOC content at the end of incubation experiment (g kg^–1^), *a* is the overall reduction in soil DOC content during the whole incubation period (g kg^–1^), and *k* is an empirical constant of DOM decomposition.

### 2.3. Characterization of DOM

The DOM of shredded straw was extracted using the method described by Hu [31]. In brief, 2 g of soil sample or 0.5 g of shredded straw sample was placed in a 50 mL polypropylene centrifuge tube and extracted with ultrapure Milli-Q water at a solid/liquid ratio of 1/10 (soil) of 1/20 (straw) (*w*/*v*) on a shaker for 24 h at 220 rpm and 25 °C. Subsequently, the sample was centrifuged at 4000 rpm for 10 min, and then the supernatant was filtered through a 0.45 μm membrane filter to obtain the filtrate, which was stored at 4 °C prior to analysis.

Fluorescence EEM was determined for the samples of DOM extract with an Aqualog spectrofluorometer (Horiba JY, Edison, NJ, USA), with excitation and emission wavelength ranges of 200–460 nm and 270–600 nm, respectively [32,33]. For each sample, background noise was eliminated using the EEM spectrum of a MilliQ water blank. UV absorbance was measured simultaneously to remove inner filter effects. Rayleigh and Raman scattering lines in the EEM spectrum were also removed. For each sample, parallel factor analysis (PARAFAC) was performed with SOLO (Eigenvector Research Inc., Manson, WA, USA) to identify the fluorescent components, which were quantified in terms of the maximum fluorescence intensity (*F*_max_) [33]. DOC content of the water extract was analyzed with a total organic carbon analyzer (Aurora 1030W, OI Analytical, College Station, TX, USA).

### 2.4. Geochemical Fractionation of Cd in Soil

Geochemical fractions of Cd in soils were analyzed using the method of Tessier et al. [34]. Then, 1 g of soil was placed in a 50 mL polypropylene centrifuge tube and weighed, then the sequential extractions were conducted for exchangeable (1 M of MgCl_2_, pH = 7), carbonate-bound (1 M of NaOAc, pH = 5), Fe-Mn oxide-bound (0.04 M of NH_2_OH·HCl in 25% (*v*/*v*) HOAc, pH = 2, and heated at 96 °C), organic matter-bound (0.02 M HNO_3_ in 30% H_2_O_2_, pH = 2), and residual (digested with 3:1 concentrated HCl/HNO_3_) fractions. The concentration of Cd in the extract was determined by an inductively coupled plasma-mass spectrometer (NexION^TM^300, PerkinElmer, Waltham, MA, USA).

### 2.5. Fluorescent Indices 

Three fluorescent indices, including fluorescent index (FI), humification index (HIX), and biological index (BIX), were calculated based on the EEM spectra, according to the common methods [15,35,36,37].

FI was the ratio of emission intensity (I) at 450 nm to that at 500 nm at an excitation of 370 nm (Equation (2)) [38]. FI has been widely used to identify the source of dissolved organic matter in ocean, coastal, fluvial, and lake ecosystems, and wastewater treatment plant effluent [39,40].

(2) $$FI=I_{\mathrm{Em}450}/I_{\mathrm{Em}500}\left(\lambda_{\mathrm{Em}370}\right)$$

HIX was calculated as the ratio of the integrated emission intensity at 435–480 nm to the sum of the integrated emission intensities at 300–345 nm and 435–480 nm at an excitation of 254 nm (Equation (3)) [36]. HIX was used to quantify the relative degree of soil humification based on the theory that the decomposition and humification of soil organic matter will lead to a lower H:C ratio and a red shift on EEM spectra.

(3) $$HIX=\sum I_{\mathrm{Em}435-480}/\left(\sum I_{\mathrm{Em}300-345}+\sum I_{\mathrm{Em}435-480}\right)\left(\lambda_{\mathrm{Ex}254}\right)$$

BIX was used to decipher the presence of autochthonous biological organic matter and was operationally calculated by dividing the emission intensity of *β* fluorophore (at 380 nm) by that of *α* fluorophore (at 430 nm) at an excitation of 310 nm (Equation (4)). The BIX was also referred as *β*:*α* in some other studies [41].

(4) $$\mathrm{BIX}=I_{\mathrm{Em}380}/I_{\mathrm{Em}430}\left(\lambda_{\mathrm{Ex}310}\right)$$

### 2.6. Other Physical and Chemical Analyses

Soil organic matter (SOM) was determined using a TOC analyzer (TOC-V-SSM5000A, Shimadzu, Tokyo, Japan). Soil pH was measured in distilled water at a soil-to-water ratio of 1:2.5 (*w*/*v*) with a pH meter (PHS-3C, INESA Instrument, Shanghai, China) [28]. The decomposition of straw was determined as mass loss of the straw in a mesh bag by oven-drying and weighing.

### 2.7. Statistical Analysis

The data were statistically analyzed by one-way analysis of variance (ANOVA). The significance of the difference between means of different treatments was tested using R 3.4.4 (R Foundation for Statistical Computing, Vienna, Austria).

## 3. Results

### 3.1. Dynamics of Total and Dissolved Organic Matter Content in Soil

The detected DOC contents in the experimental soils were the result of different processes including leaching release of soluble organic compounds from straw, decomposition of straw, sorption of DOM to soil particles, and decomposition and transformation of DOM [17,42]. In all the soils, organic matter content decreased rapidly in the first two weeks but slowly afterwards. The decreases in organic matter content with time during the initial stage of incubation were greater in the straw amended soils than in the control soils, irrespective of straw type (Figure S1). The temporal trends of soil organic matter content were in accordance with the temporal changes of cumulative decomposition observed for the straw in mesh bags buried in the soils (i.e., rapid increases in the first two weeks followed by slow increases afterwards) (Figure S2). It was found that aerobic (unsaturated) conditions could favor the decomposition of both maize and rice straw in the soils, which is in agreement with the findings of many previous studies [43,44,45]. The decomposition of crop straw is more dependent on soil moisture conditions than on soil properties [46]. Inconsistent effects of moisture changes on the decomposition of organic matter in soils have been documented for different organic amendments. For instance, during the rewetting of the more intensely dried soils, a lower decomposition rate of rice straw was observed [47], but a higher decomposition rate of amended litter was reported [48]. In addition, most DOM, once leached from straw or desorbed from the soil, could be mineralized shortly (e.g., 7 days) in the aqueous phase [49,50].

**Figure 2:**
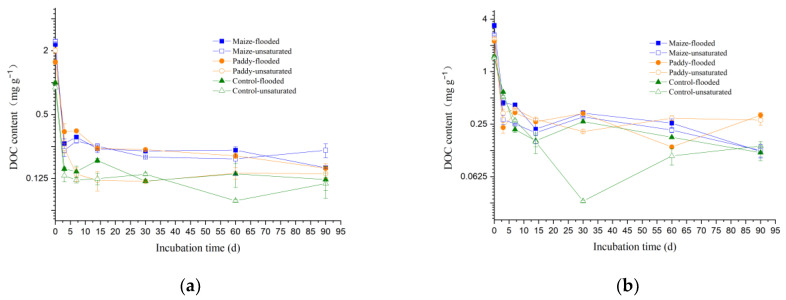
Temporal changes of dissolved organic matter content (represented by dissolved organic carbon (DOC)) in the soils collected from sites A (**a**) and B (**b**) during laboratory incubation with/without straw addition under flooded/unsaturated conditions. The Y ordinate is logarithmically transformed (log2).

The effects of straw type and moisture condition on soil DOM were revealed through laboratory incubation experiments. Temporal changes in DOC content in the soils under different treatments are presented in Figure 2. The content of DOC in both soils, irrespective of moisture condition and straw addition, decreased quickly from day 0 to day 3, declined slowly from day 3 to day 14, and remained at a nearly constant level after day 14. DOC content was significantly higher (*p* < 0.05) in the straw amended soils than in the control soils, and this difference gradually decreased with time during the incubation. Initial DOC content was higher for the soil from site B than for the soil from site A, regardless of experimental treatments. After day 14 of incubation, the difference in DOC content between the soils from the two sites became insignificant for all four treatments with straw addition (*p* > 0.05) but remained significant for the controls (*p* < 0.05). First-order kinetic equations can be fitted satisfactorily (*p* < 0.05) to the time series data of DOC content in all experimental soils (Table 1). The observed highest DOC contents at the beginning of the incubation (i.e., day 0) for all soil samples reflect that air-drying treatment can increase the amount of DOM in soil. This agrees with previous findings in other soils that DOC release from air-dried soil was larger than that from moist soil [12,15,51,52]. Straw return to the farmland resulted in higher DOC contents on day 0, when compared to the control. The air-drying induced elevation of DOC content on day 0 was of a greater magnitude in the soil with maize straw return (by 173 ± 7% relative to the control) than in the soil with rice straw return (by 123 ± 5% relative to the control). 

In the control soils without straw addition, the quick (*a* = 0.788–1.322, *k* = 0.371–1.417) decrease in DOM level within the first three days of the incubation could be attributed mainly to the increase in sorption of DOM to soil particles with time after rewetting treatment. The more dramatic decline (*a* = 1.322–3.115, *k* = 0.794–14.505) in DOM level in the straw amended soils at the initial stage of the incubation could be attributed to the decomposition, sorption, and mineralization of the straw-derived DOM, which had different compositions from that in the control soil (described later). In our incubation system without drainage, the rapid decrease in DOC content within the first three days can be attributed to the sorption of DOM to soil particles and biotic or abiotic decomposition of DOM [31,49].

### 3.2. Composition of DOM Fluorescent Components

By PARAFAC analysis of the EEM spectra across water extracts of time series soil samples for each straw addition treatment, a total of three fluorescent components, including component 1 (C1) at 280/340 nm (Ex/Em), component 2 (C2) at 200 (300)/400 nm (Ex/Em), and component 3 (C3) at 260 (360)/450 nm (Ex/Em), were identified. C1 represents a tryptophan-like/protein-like substance (i.e., peak T defined in Coble et al. [53]) and reflects the microbial activity and bioavailability of DOM [54,55]. C1 usually constitutes the biodegradable part of DOM and has simple molecular structures. C2 represents UV humic-like and visible marine humic-like substance (i.e., peaks A and M defined in Coble et al. [53]). C3 represents UV humic-like and visible humic-like substance (i.e., peaks A and C defined in Coble et al. [56]). C2 and C3 resemble microbial oxidized components and have abundant aromatic structures and high molecular weight [57]. Similar compositions of DOM in different farmland soils were reported by a number of previous studies [17,58,59].

**Figure 3:**
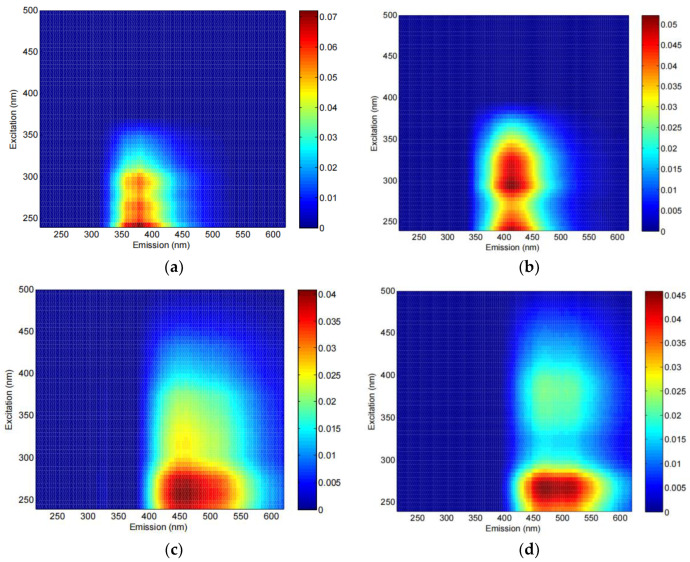
EEM locations, representative EEMs, and spectral loadings of fluorescent components in straw-derived DOM identified by PARAFAC: (**a**) C2 in rice straw amended soils; (**b**) C2 in the maize straw amended soils; (**c**) C3 in the rice straw amended soils; (**d**) C3 in the maize straw amended soils. Values of each EEM spectrum represent all the samples collected across the whole incubation period for a specific straw addition treatment.

C1 was found in all the straw amended soils but not in the control soils, indicating that the addition of straw introduced a new tryptophan-like/protein-like component to the soils. Differences in the composition of C2 were observed between the two straw treatments: peak M was detected in the treatment with maize straw but not in the treatment with rice straw (Figure 3). The primary and secondary fluorescence peaks of C3 were red-shifted from 450 nm (Em) in the rice straw amended soils to 500 nm (Em) in the maize straw amended soils (Figure 3). For a soil DOM component, a red shift in fluorescence maxima indicates that it becomes has a more complex structure [53].

**Figure 4:**
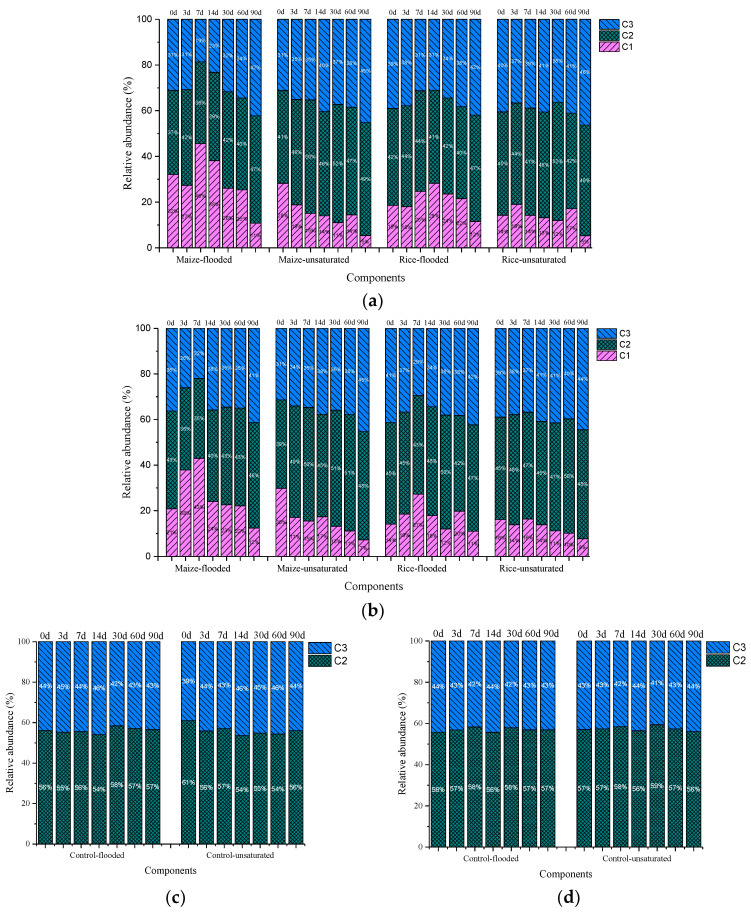
Temporal changes of relative abundances of fluorescent DOM components in the soils under different treatments during laboratory incubation: straw amended soil of site A (**a**); straw amended soil of site B (**b**); control soil of site A (**c**); control soil of site B (**d**).

Relative abundances of the three fluorescent components were calculated to evaluate temporal changes in DOM composition as affected by straw addition (Figure 4). The relative abundance of C1, C2, and C3 varied in the range of 0–46%, 35–61%, and 19–46%, respectively. Generally, the relative abundance of C1 showed high values in the first one or two weeks and then decreased in the straw amended soils. The amendment of maize straw led to higher relative abundances of C1 when compared to the amendment of rice straw. Overall, relative abundances of C1 in the soils under flooded conditions were higher than those under unsaturated conditions. C2 and C3 exhibited gradual increasing trends with time in the straw amended soils. In the control soils, the relative abundance of these two components remained constant over time, with that of C2 being higher than that of C3.

Fluorescence indexes of DOM in the soils from both sites followed the same trend of temporal changes (Tables S1 and S2). HIX was higher in the control soils than the straw amended soils, and exhibited marked increases with time in the latter to values close to those in the control soils; unsaturated condition led to higher HIX than flooded condition, reflecting that aerobic condition favored the humification of DOM. Both FI and BIX showed decreasing temporal changes in the straw amended soils, but such trends were not observed in the control soils. The detected FI values across all experimental soils (1.10–1.34) are in the reported range for terrestrial or allochthonous DOM sources [35]. FI in the straw amended soils were slightly higher than those in the controls soils at the early stage of incubation, whereas such effect of straw return disappeared at the later stage. The observed decrease in BIX with time in the straw amended soils indicates that the albuminoid and biological components dissipated during the course of straw decomposition [41].

### 3.3. Dynamics of Cd Speciation in Soil

**Figure 5:**
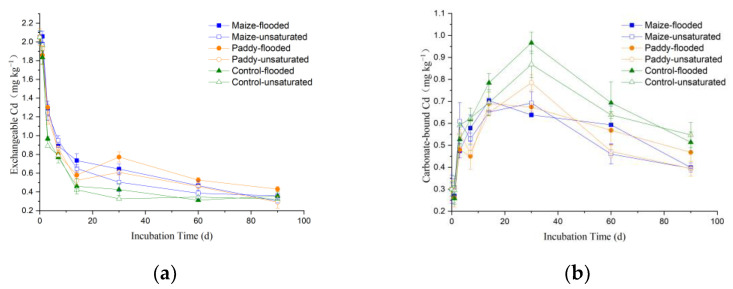

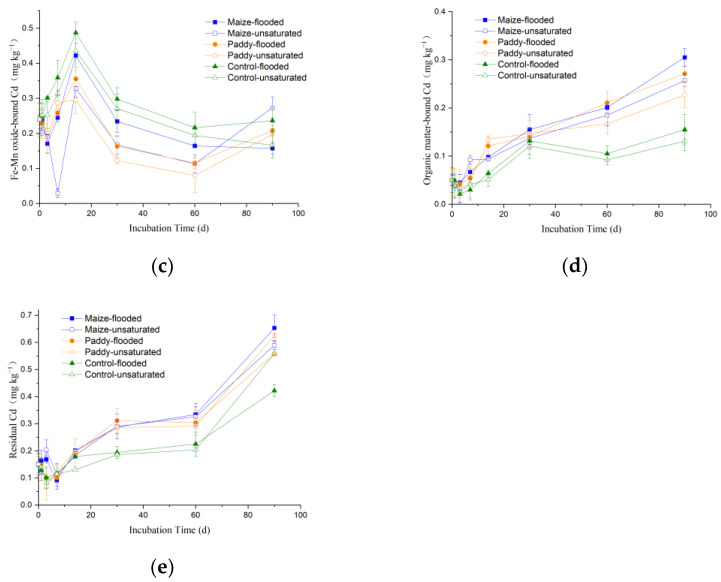
Temporal changes of the content of different Cd fractions (exchangeable Cd (**a**), carbonate-bound Cd (**b**), Fe-Mn oxide-bound Cd (**c**), organic matter-bound Cd (**d**), and residual Cd (**e**)) in the soil collected from site A of high pollution levels during laboratory incubation under different treatments.

**Figure 6:**
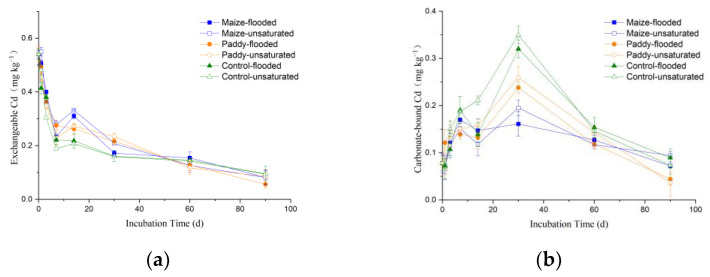

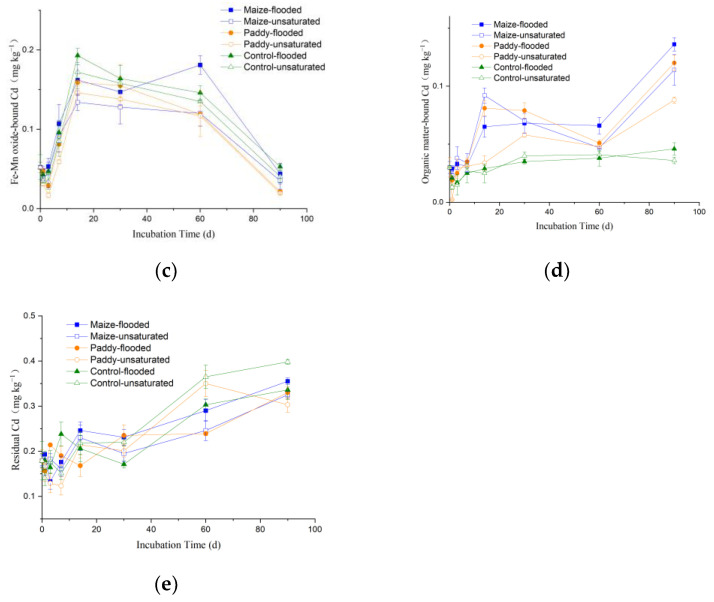
Temporal changes of the content of different Cd fractions (exchangeable Cd (**a**), carbonate-bound Cd (**b**), Fe-Mn oxide-bound Cd (**c**), organic matter-bound Cd (**d**), and residual Cd (**e**)) in the soil collected from site B of low pollution level during laboratory incubation under different treatments.

The temporal changes of Cd speciation in the soils under different treatments are shown in Figure 5 and Figure 6. At the beginning of the laboratory incubation experiment, the percentage of Cd geochemical fractions in the soil collected from site B (of low pollution level) followed the order: exchangeable Cd > residual Cd > carbonate-bound Cd > Fe-Mn oxide bound Cd > organic matter-bound Cd, with the exchangeable fraction accounting for 58% of total Cd content (Figure S3). With increasing incubation time, the content of different Cd fractions in the soil of site B showed different temporal change patterns: the exchangeable Cd decreased rapidly in the first week and then slowly afterwards (Figure 6a); the carbonate and Fe-Mn oxide-bound Cd first increased and then decreased, with the maxima occurring on days 30 and 14, respectively (Figure 6b,c); the organic matter-bound and residual Cd gradually increased (Figure 6d,e). At the early stage of incubation, compared with the control soil (without straw) of site B, the addition of straw led to increases in exchangeable and organic matter-bound Cd but decreases in carbonate and Fe-Mn oxide-bound Cd (Figure 6).

Initial Cd speciation in the soil collected from site A (of high pollution level), which was slightly different from that (in percentage) of site B, follows the order: exchangeable Cd > carbonate-bound Cd > Fe-Mn oxide-bound Cd > residual Cd > organic matter-bound Cd, with the exchangeable fraction accounting for a greater percentage (73%) of total Cd content (Figure S3). The higher exchangeable Cd content in the soil at site A indicates that most Cd came from exogenous input and was mainly sorbed on soil particles through electrostatic attraction. With the passage of incubation time, the five Cd fractions of the soil from site A exhibited the same temporal changes as those of the soil from site B. In the incubated samples of the soil from site A, the exchangeable Cd decreased rapidly in the first two weeks and then slowly afterwards (Figure 5a); the carbonate and Fe-Mn oxide-bound Cd first increased and then decreased, with the peaks occurring on days 30 and 14, respectively (Figure 5b,c); the organic matter-bound and residual Cd increased gradually (Figure 5d,e). The effects of adding straw on Cd speciation, except for the residual Cd, in the soil from site A were the same as those in the soil from site B. Notably, the residual Cd content in the soil from site A was increased by straw return; in contrast, no significant effects of straw return on residual Cd were observed for the soil from site B. After 90 days of incubation, the sum (in percentage) of carbonate and Fe-Mn oxide Cd in the soil from site A was significantly higher than that of the soil from site B (*p* < 0.01), while the sum of the two least available Cd forms (i.e., organic and residual Cd) was significantly lower than that of the soil from site B (Figure S3).

Carbonate and Fe-Mn oxide-bound Cd are moderately active forms between an exchangeable and residual fraction; therefore, they both experienced an increase in response to a sharp decrease in exchangeable Cd at the early stage of incubation (Figure 5a and Figure 6a). After 7 or 14 days, as the exchangeable Cd content tended to be stable, carbonate and Fe-Mn oxide-bound Cd continued to undertake transformation into the two least labile forms (i.e., organic matter-bound and residual Cd). The effects of straw addition on Cd speciation eventually became weaker at the end of incubation, as also noted by previous studies [12]. Apparently, flooding treatment promoted the transformation of exchangeable and carbonate-bound Cd into organic matter-bound and residual Cd. After 90 days of incubation, the sum (in percentage) of organic matter-bound and residual Cd in the soils under flooded conditions was significantly higher than that under unsaturated conditions (Figure S3). In addition, no significant differences in straw return effect on soil Cd speciation were observed between maize and rice straw (*p* > 0.05).

## 4. Discussion

### 4.1. Effects of Straw Return and Soil Moisture on Soil DOM Content

The decomposition of straw in soils is affected by many factors, among which moisture and aeration are the main ones [46]. During the early stage of straw decomposition, a large amount of soluble organic compounds and inorganic mineral nutrients released into soils may improve the growth of microbes, and thus, lead to enhanced biological decomposition of straw. The easily decomposable components in the straw decrease with time while the refractory components gradually accumulate, leading to decreases in biological activity and the rate of microbe-meditated straw decomposition [43,60]. The aerobic conditions may favor the decomposition of straw in soils. Under unsaturated conditions, soils contain sufficient amounts of both oxygen and water, which is conducive to microbial respiration and straw degradation, while saturated conditions may inhibit the activity of aerobic microbes in soils, resulting in a low respiration intensity of microbes and a slow decomposition rate of straw organic matter [47]. The vulnerable straw components, which can account for 50–90% of the straw mass and show distinct organic matter composition (higher contents of proteinaceous, amino, and aliphatic compounds) from that in pristine soil [61], can be released into soils, and thus, lead to substantial increases in soil DOM in 3 to 4 months through leaching and decomposition [14].

Straw amendment and soil moisture conditions can affect the DOM content in soils. The initial increases in soil DOC content observed in this study came mainly from the decomposition of straw. At the later stage, the readily degraded DOM in the straw was depleted rapidly, as indicated by the observed quick decline of soil DOC to a nearly constant level. The stable DOC level in soils after day 14 of the incubation derived largely from the decomposition of soil humus. In addition, the decrease in DOC with time may be partly attributed to the sorption of DOM by the soil through electrostatic adsorption, ligand exchange-surface complexation, hydrophobic interaction, entropy effect, hydrogen bonding, cationic bond bridging, and/or the formation of insoluble organic matter through complexation, chelation, flocculation, and precipitation [62]. The release of organic matter upon wetting was dominated initially by microbial sources (e.g., dead microbes, extracellular polymeric substrates, and intracellular compounds) and later by non-microbial sources (e.g., organic matter bound to clay minerals, plant debris, and root exudates) [63,64,65,66]. Moreover, wetting of dried soil is known to lead to a decrease in soil water repellency with time [67], and thus, could increase the accessibility of heterogeneous soil on the micro-scale (µm to mm) and consequently favor the redistribution of DOM between the solid and aqueous phase of the soil. Sorption of DOM to soil is selective for hydrophobic and aromatic compounds [35,51]. DOM enriched with aromatic or hydrophobic compounds, probably derived from lignin, can be preferentially sorbed by soil and, thus, stabilized [49]. Decreases in degradation of simple organic compounds (e.g., glucose, citrate, oxalate, and malate) after sorption to soils were reported [68,69,70]. However, to date, there has been little quantitative information about the decomposition and transformation of DOM sorbed on soil particles.

### 4.2. Effects of Straw Return on Cd Speciation in Soil

Cd can react with DOM through the formation of soluble complexes and with soil solids (mainly clay silicates, oxides, and humus) through specific and non-specific sorption. DOM is a potential extractant for Cd. The extraction of Cd by DOM is dependent on the concentration and composition of DOM, soil sorbing characteristics, and environmental factors and conditions [71]. The introduction of additional DOM derived from straw can cause distinctive changes in Cd speciation in soil.

Temporal changes of Cd observed in this study can be attributed to the decomposition dynamics of organic matter in the experimental soils. Decomposition of products of straw at the early stage of incubation could cause changes in soil properties, which in turn led to the variations in the distribution of Cd geochemical fractions in the soils with time. Correlation analysis between Cd fractions and DOC content throughout the incubation period and across the experimental soils provides some useful information about the potential of DOM in modulating Cd fraction distribution. A significant linear positive correlation was observed between exchangeable Cd and DOC content (*p* < 0.05, R^2^ = 0.826). The markedly higher contents of exchangeable Cd at the early stage of incubation than at the later stage can be attributed to the higher DOM levels in all the treatments. The DOM derived from straw is usually rich in functional groups with high degrees of unsaturation such as carboxyl, aldehyde, and ketone groups [72]. These functional groups are more reactive in DOM than in solid organic matter and can complex/chelate Cd to form organometallic complexes. As a carrier of Cd, DOM can enhance the activity of Cd and promote the dissolution of Cd in soil. Similarly, decreases in exchangeable forms of metals with time were previously reported in other soils, which were attributed to transformation to stable forms during aging [73].

The observed lower final percentage contents of organic matter-bound and residual Cd in the straw amended soils from heavily polluted site A may be attributed to two reasons: (1) the higher content of Cd in the soil from site A may more strongly inhibit the activity of microbes to decompose the straw, and thus, limit to a greater extent the development of the soil’s affinity to Cd with straw decomposition; (2) given the same straw addition rate, the supply of additional binding sites induced by straw addition in the soil from site A could be less sufficient.

### 4.3. Correlations among Soil Cd Speciation and DOM Fluorescence Parameters

**Figure 7:**
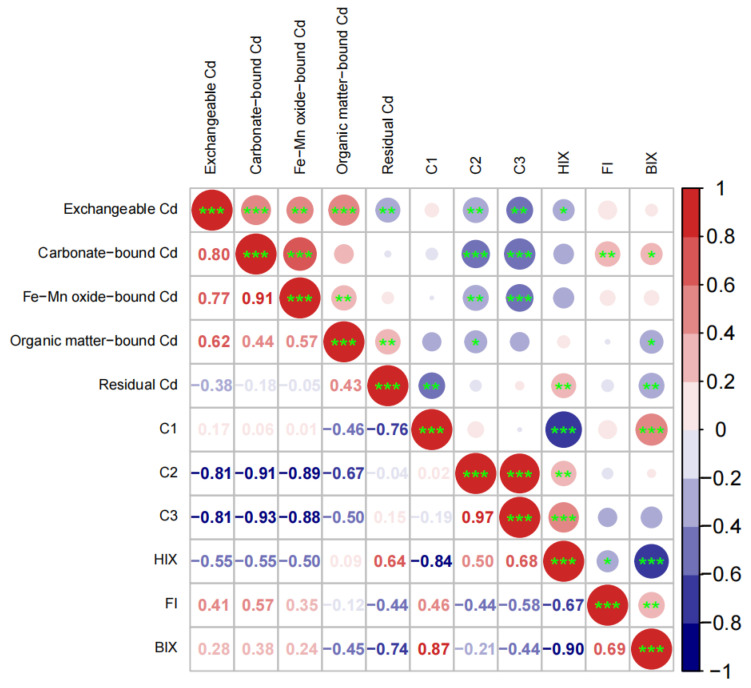
Correlations among the Cd chemical fractions, the intensities of fluorescent components, and the fluorescence indices (*, **, and *** represent 0.05, 0.01, and 0.001 probability level of significance, respectively).

Results of correlation analysis among the five Cd geochemical fractions, the intensities of the three fluorescent components and the three fluorescence indices are presented in Figure 7. The fluorescence intensity of C1 was negatively correlated with residual Cd, while the fluorescence intensity of C2 and C3 was negatively correlated with exchangeable, carbonate, and Fe-Mn oxide-bound Cd (*p* < 0.01). It can be inferred that the variations of the three weakly bound Cd fractions (i.e., exchangeable, carbonate, and Fe-Mn oxide-bound Cd), during laboratory incubation eventually lead to the increase in the most strongly bound Cd fraction (i.e., residual Cd), which is a complex process facilitated by Cd association with the two humified components (i.e., C2 and C3). A previous study using fluorescence quenching showed that fulvic-like substance (at 340/470 nm (Ex/Em)) could have a stronger and more stable interaction with Cd than tyrosine-like substance (at 275/300 nm (Ex/Em)) [74]. Therefore, the observed transformation of Cd from labile forms into residual forms during the incubation can be partly attributed to the increase in C2 and C3 abundance with time.

This mechanism proposed in this study is supported by the observed positive correlation of HIX with residual Cd (*p* < 0.01). There was a very significant negative relationship between HIX and BIX (*p* < 0.001) and accordingly a negative correlation of BIX with residual Cd was observed (*p* < 0.01). In addition, FI was positively correlated with carbonate-bound Cd (*p* < 0.01) (Figure 7). In a previous study, a negative correlation of HIX with BIX was also observed; however, a positive correlation of HIX with FI was found [75], which is contrary to our results (Figure 7). This inconsistency may result from differences in not only incubation conditions but also soil and straw type. According to a recent soil survey in the Zhangxi watershed, exchangeable Cd (extracted with CaCl_2_) exhibited a weakly positive correlation with FI but a weakly negative correlation with HIX [76]. Therefore, we propose that DOM fluorescence parameters can be considered and selected for predicting Cd geochemical fractions in soil that are apparently meditated by DOM.

## 5. Conclusions

A 90-d laboratory incubation experiment was conducted to evaluate the effects of straw return and moisture condition on temporal changes of DOM composition and Cd speciation in paddy-upland rotation farmland soils. It was found that straw addition substantially increased soil DOM content in the first three days. The application of straw introduced a protein-like fluorescent component of DOM, and the relative abundance of this unique component generally showed moderate declining trends over time in straw amended soils except for slight increases in the first week under flooded conditions. The sum of two strongly bound Cd forms (i.e., organic matter-bound and residual fraction) increased with incubation time in straw amended soils, and such effect was more significant under flooded conditions. During straw decomposition, the humified components (C2 and C3) of DOM were negatively correlated to the three weakly bound Cd (i.e., exchangeable, carbonate-bound, and Fe-Mn oxide-bound Cd) (*p* < 0.01) while its protein-like component (C1) was negatively correlated with the most strongly bound residual Cd (*p* < 0.01). Apparently, the fluorescent components may have strong impacts on Cd speciation during straw decomposition process in soil and selected fluorescence parameters have a potential for predicting temporal changes of Cd geochemical fractions. In Cd-polluted farmland, flooding in the first three days after straw return should be avoided to reduce the risk of DOM-facilitated Cd release through leaching.

## Figures and Tables

**Table 1:** Fitted equations to the laboratory incubation experimental data of temporal changes of soil DOM content.

Soil Sampling Site	Straw Type	Moisture Condition	DOC Content ^1^	R^2^
			Fitted Equation	
A	Maize	Unsaturated	$C_{t}=0.230+2.232e^{-1.031t}$	0.998 * ^1^
		Flooded	$C_{t}=0.232+2.070e^{-1.352t}$	0.995 *
	Rice	Unsaturated	$C_{t}=0.130+1.877e^{-0.942t}$	0.999 *
		Flooded	$C_{t}=0.234+1.322e^{-0.794t}$	0.980 *
	Control	Unsaturated	$C_{t}=0.114+0.788e^{-1.241t}$	0.994 *
		Flooded	$C_{t}=0.141+0.856e^{-1.417t}$	0.993 *
B	Maize	Unsaturated	$C_{t}=0.218+2.422e^{-1.211t}$	0.995 *
		Flooded	$C_{t}=0.267+3.115e^{-0.952t}$	0.991 *
	Rice	Unsaturated	$C_{t}=0.287+2.093e^{-1.256t}$	0.994 *
		Flooded	$C_{t}=0.306+1.629e^{-14.505t}$	0.941 *
	Control	Unsaturated	$C_{t}=0.116+1.322e^{-0.371t}$	0.986 *
		Flooded	$C_{t}=0.175+1.319e^{-0.404t}$	0.986 *

^1^ * represents 0.05 probability level of significance (two-tailed).

## Data Availability

The data presented in this study are available from the corresponding author upon request.

## Supplementary Materials

The following supporting information can be downloaded at: https://www.mdpi.com/article/10.3390/ijerph191912128/s1, Figure S1: Temporal changes of organic matter content in the soils (collected from sites A (a) and B (b)) with or without thorough mixing with straw during laboratory incubation under different moisture conditions; Figure S2: Temporal changes of cumulative decomposition of straws in mesh bags buried in the soils collected from sites A (a) and B (b) during laboratory incubation under different treatments; Figure S3: Temporal changes of Cd fraction distribution in the soils collected from sites A (a) and B (b) during laboratory incubation under different treatments; Table S1: Fluorescence indices of dissolved organic matter in the soils collected from site A at different times during laboratory incubation under different treatments (mean ± standard deviation); Table S2: Fluorescence indexes of dissolved organic matter in the soils collected from site B at different times during laboratory incubation under different treatments (mean ± standard deviation).
