# Supplementary material for: Effects of Straw Return and Moisture Condition on Temporal Changes of DOM Composition and Cd Speciation in Polluted Farmland Soil
Source: Int J Environ Res Public Health. 2022 Sep 25;19(19):12128. doi: 10.3390/ijerph191912128 (PMC9566551; doi:10.3390/ijerph191912128)
Supplement: Supplementary file 1 [file ijerph-19-12128-s001.zip › ijerph-1910443-supplementary.pdf]

Supplementary Information

Effects of Straw Return and Moisture Condition on Temporal Changes of DOM Composition and Cd Speciation in Polluted Farmland Soil

Guang Yang [1,2], Xiangyu Tang [1,3,*], Zhuo Guan [1] and Junfang Cui [1]

[1] Key Laboratory of Mountain Surface Processes and Ecological Regulation, Institute of Mountain Hazards and Environment, Chinese Academy of Sciences, Chengdu 610041, China
[2] University of Chinese Academy of Sciences, Beijing 100049, China
[3] State Key Laboratory of Subtropical Silviculture, Zhejiang A&F University, Hangzhou 311300, China
* Correspondence: xytang@imde.ac.cn

Number of figures: 3

Number of tables: 2

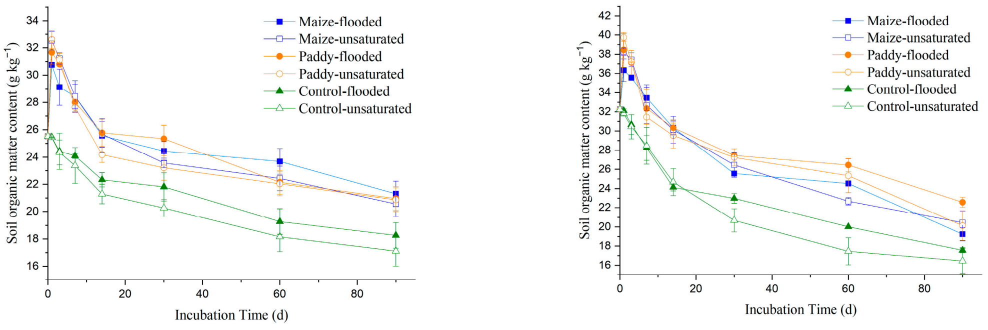

(**a**) (**b**)

Figure S1. Temporal changes of organic matter content in the soils (collected from sites A (**a**) and B (**b**)) with or without thorough mixing with straw during laboratory incubation under different moisture conditions.

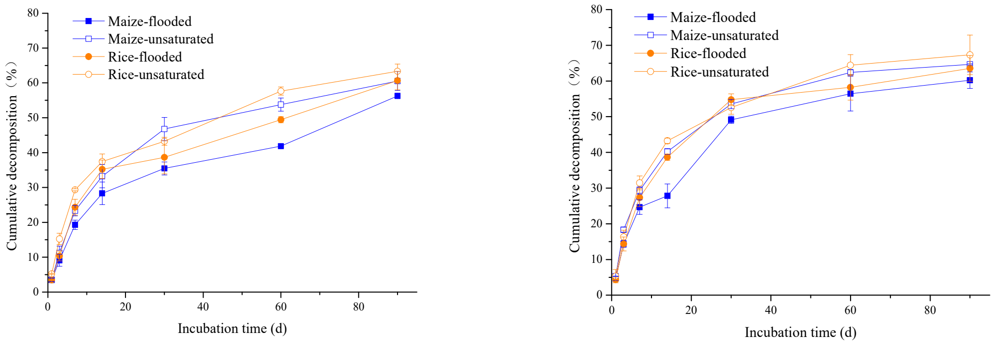

(**a**) (**b**)

Figure S2. Temporal changes of cumulative decomposition of straws in mesh bags buried in the soils collected from sites A (**a**) and B (**b**) during laboratory incubation under different treatments.

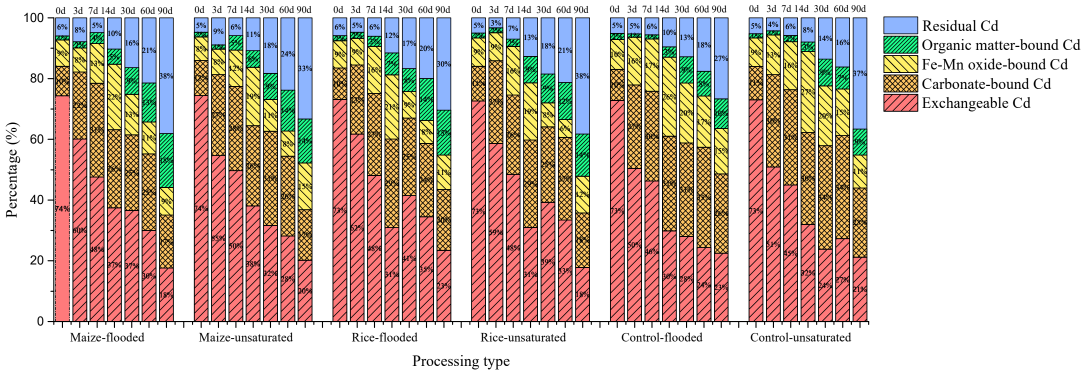

(**a**)

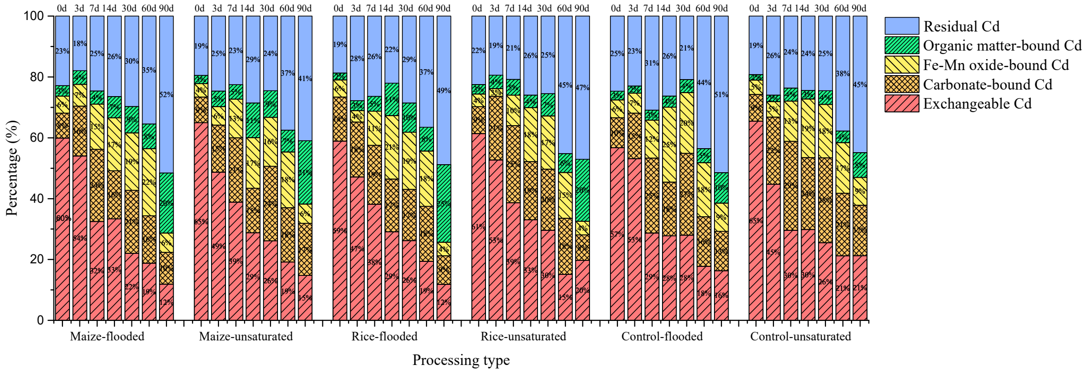

(**b**)

Figure S3. Temporal changes of Cd fraction distribution in the soils collected from sites A (**a**) and B (**b**) during laboratory incubation under different treatments.

Table S1. Fluorescence indices of dissolved organic matter in the soils collected from site A at different times during laboratory incubation under different treatments (mean±standard deviation).

Incubation time (d)	Treatments	HIX	FI	BIX
0	Maize-flooded	0.81±0.03	1.19±0.16	0.62±0.04
	Maize-unsaturated	0.81±0.10	1.34±0.11	0.68±0.00
	Paddy-flooded	0.87±0.09	1.18±0.09	0.57±0.01
	Paddy-unsaturated	0.89±0.09	1.21±0.15	0.57±0.06
	Control-flooded	0.92±0.07	1.16±0.11	0.54±0.07
	Control-unsaturated	0.89±0.04	1.18±0.12	0.65±0.10
3	Maize-flooded	0.81±0.06	1.33±0.02	0.75±0.10
	Maize-unsaturated	0.86±0.03	1.32±0.20	0.69±0.09
	Paddy-flooded	0.87±0.07	1.21±0.17	0.63±0.06
	Paddy-unsaturated	0.85±0.07	1.23±0.08	0.65±0.08
	Control-flooded	0.93±0.07	1.16±0.03	0.57±0.08
	Control-unsaturated	0.91±0.06	1.19±0.17	0.58±0.04
7	Maize-flooded	0.70±0.01	1.31±0.15	0.86±0.01
	Maize-unsaturated	0.89±0.02	1.34±0.08	0.70±0.10
	Paddy-flooded	0.83±0.08	1.29±0.10	0.68±0.07
	Paddy-unsaturated	0.90±0.04	1.24±0.15	0.63±0.06
	Control-flooded	0.95±0.10	1.15±0.13	0.55±0.01
	Control-unsaturated	0.94±0.02	1.17±0.12	0.58±0.02
14	Maize-flooded	0.75±0.02	1.28±0.20	0.72±0.07
	Maize-unsaturated	0.90±0.02	1.24±0.07	0.63±0.01
	Paddy-flooded	0.80±0.07	1.26±0.16	0.65±0.09
	Paddy-unsaturated	0.90±0.10	1.23±0.13	0.62±0.08
	Control-flooded	0.92±0.07	1.13±0.10	0.52±0.06
	Control-unsaturated	0.94±0.06	1.18±0.05	0.54±0.10
30	Maize-flooded	0.84±0.10	1.33±0.18	0.68±0.05
	Maize-unsaturated	0.93±0.04	1.29±0.11	0.69±0.08
	Paddy-flooded	0.84±0.07	2.04±0.13	0.65±0.06
	Paddy-unsaturated	0.91±0.07	1.33±0.17	0.67±0.05
	Control-flooded	0.90±0.07	1.18±0.00	0.57±0.04
	Control-unsaturated	0.95±0.09	1.17±0.16	0.57±0.04
60	Maize-flooded	0.82±0.10	1.18±0.08	0.64±0.06
	Maize-unsaturated	0.88±0.08	1.22±0.02	0.66±0.09
	Paddy-flooded	0.84±0.02	1.18±0.01	0.59±0.05
	Paddy-unsaturated	0.88±0.02	1.15±0.02	0.53±0.07
	Control-flooded	0.83±0.07	1.16±0.10	0.63±0.09
	Control-unsaturated	0.83±0.06	1.15±0.17	0.55±0.00
90	Maize-flooded	0.91±0.08	1.21±0.02	0.61±0.01
	Maize-unsaturated	0.91±0.05	1.19±0.10	0.55±0.02

Paddy-flooded	0.91±0.06	1.19±0.13	0.59±0.01
Paddy-unsaturated	0.95±0.08	1.15±0.01	0.52±0.01
Control-flooded	0.93±0.03	1.18±0.19	0.58±0.01
Control-unsaturated	0.95±0.04	1.16±0.06	0.54±0.02

Table S2. Fluorescence indexes of dissolved organic matter in the soils collected from site B at different times during laboratory incubation under different treatments (mean±standard deviation).

Incubation Time (d)	Treatments	HIX	FI	BIX
0	Maize-flooded	0.86±0.07	1.17±0.03	0.61±0.04
	Maize-unsaturated	0.81±0.05	1.26±0.03	0.65±0.04
	Paddy-flooded	0.90±0.03	1.15±0.15	0.57±0.10
	Paddy-unsaturated	0.89±0.02	1.19±0.16	0.61±0.05
	Control-flooded	0.93±0.02	1.13±0.19	0.55±0.02
	Control-unsaturated	0.92±0.04	1.14±0.10	0.58±0.00
3	Maize-flooded	0.75±0.06	1.28±0.14	0.74±0.02
	Maize-unsaturated	0.87± 0.06	1.30±0.03	0.71±0.05
	Paddy-flooded	0.87±0.08	1.19±0.09	0.62±0.01
	Paddy-unsaturated	0.90±0.04	1.21±0.11	0.62±0.08
	Control-flooded	0.93±0.10	1.15±0.08	0.59±0.05
	Control-unsaturated	0.94±0.06	1.19±0.16	0.60±0.06
7	Maize-flooded	0.66±0.02	1.30±0.06	0.82±0.08
	Maize-unsaturated	0.89±0.04	1.27±0.18	0.68±0.02
	Paddy-flooded	0.81±0.08	1.25±0.14	0.73±0.04
	Paddy-unsaturated	0.89±0.07	1.19±0.01	0.61±0.09
	Control-flooded	0.94±0.05	1.15±0.00	0.58±0.04
	Control-unsaturated	0.94±0.04	1.17±0.02	0.60±0.06
14	Maize-flooded	0.83±0.03	1.17±0.12	0.60±0.06
	Maize-unsaturated	0.88±0.03	1.22±0.13	0.65±0.03
	Paddy-flooded	0.86±0.02	1.34±0.19	0.73±0.09
	Paddy-unsaturated	0.90± 0.01	1.15±0.02	0.61±0.08
	Control-flooded	0.92±0.09	1.16±0.19	0.58±0.03
	Control-unsaturated	0.94±0.10	1.17±0.17	0.59±0.04
30	Maize-flooded	0.85±0.03	1.20±0.02	0.64±0.04
	Maize-unsaturated	0.91±0.07	1.27±0.20	0.72±0.08
	Paddy-flooded	0.89±0.09	1.19±0.11	0.64±0.07
	Paddy-unsaturated	0.93±0.06	1.18±0.14	0.61±0.01
	Control-flooded	0.91±0.08	1.16±0.16	0.58±0.04
	Control-unsaturated	0.94±0.07	1.19±0.06	0.89±0.05
60	Maize-flooded	0.86±0.10	1.19±0.08	0.66±0.08
	Maize-unsaturated	0.92±0.06	1.23±0.11	0.69±0.08
	Paddy-flooded	0.87±0.04	1.12±0.15	0.58±0.09
	Paddy-unsaturated	0.93±0.05	1.20±0.07	0.60±0.09
	Control-flooded	0.93±0.06	1.10±0.13	0.59±0.03
	Control-unsaturated	0.86±0.02	1.14±0.13	0.56±0.08
90	Maize-flooded	0.91±0.10	1.18±0.04	0.62±0.07
	Maize-unsaturated	0.94±0.05	1.15±0.10	0.54±0.03
	Paddy-flooded	0.92±0.05	1.18±0.15	0.57±0.04

	Paddy-unsaturated	0.94±0.07	1.15±0.13	0.55±0.06
	Control-flooded	0.94±0.08	1.19±0.12	0.58±0.01
	Control-unsaturated	0.94±0.05	1.16±0.10	0.55±0.04
